# CIRSE Standards of Practice on Nephrostomy and Ureteric Stent Placement and Exchange

**DOI:** 10.1007/s00270-025-04328-9

**Published:** 2026-01-08

**Authors:** Anthony G. Ryan, Iain Irvine, Harry Bardgett, Rutger van der Meer, David Rea, Gianpaolo Carrafiello

**Affiliations:** 1 Division of Interventional Radiology, Department of Radiology, University Hospital Waterford, Ardkeen, Waterford City, Ireland; 2 Royal College of Surgeons in Ireland, Dublin, Ireland; 3 University College Cork, Cork, Ireland; 4 University Hospital Waterford, Ardkeen, Waterford City, Ireland; 5 Tallaght University Hospital, Dublin 24, Ireland; 6 West of West Yorkshire Uro-Oncology Centre, Bradford Teaching Hospitals, Bradford, UK; 7 Leiden University Medical Centre, Leiden, Netherlands; 8 Radiology Department, Children’s Health Ireland at Crumlin, Cooley Road, Dublin 12, Ireland; 9 Università Degli Studi Di Milano, Milan, Italy; 10 Unit of Radiology, Fondazione IRCCS Ca’ Granda Ospedale Maggiore Policlinico, Milan, Italy

**Keywords:** Interventional radiology, Percutaneous nephrostomy, Percutaneous ureteric stenting, Malignant ureteric obstruction, Benign ureteric obstruction, Renal tract calculi, PCNL

## Abstract

**Background:**

Obstructive uropathy is a very common pathology of the genitourinary system which, if untreated, leads to renal impairment, end-stage renal failure and death. Particularly in the case of acute obstructive uropathy, urgent decompression is necessary to prevent compression-mediated ischaemia of the renal parenchyma and the development of irreversible renal failure. Percutaneous nephrostomy is a well-established and relatively safe image-guided procedure used to obtain access to the renal collecting system and is the procedure of choice for the infected obstructed kidney, minimising the risk of septic shock and possible death. Subsequent internalisation via antegrade ureteric stenting is frequently employed to relieve obstruction at the level of the causative lesion.

**Purpose:**

CIRSE Standards of Practice documents recommend a reasonable approach to, and best practices for, performing procedures, in this instance, Nephrostomy and Ureteric Stent Placement and Exchange.

**Methods:**

The writing group, established by the CIRSE Standards of Practice Committee, consisted of five clinicians with internationally recognised expertise in this topic, and one research assistant (I.I.). The writing group reviewed the existing literature, performing a pragmatic evidence search using PubMed to search for publications in English relating to human subjects from 2001 to 2025. Relevant older primary sources were included where the data have not been updated.

**Results:**

A document was produced, making recommendations for practice based on currently available evidence in a range of clinical scenarios.

## Introduction

The CIRSE Standards of Practice (SOP) committee established a writing group which was tasked with producing up-to-date recommendations for the performance of percutaneous nephrostomy and ureteric stent placement and exchange. CIRSE SOP documents are neither clinical practice guidelines nor systematic reviews of the literature. This document is intended to recommend a reasonable approach to, and best practices for, performing these procedures, rather than impose a standard of clinical patient care. Institutions should regularly review their internal procedures for development and improvement, taking into account international guidance, local resources, and regular internal morbidity and mortality reviews. A summary of key recommendations can be found in Table 1.

**Table 1:** Summary of recommendations

	Direct calyceal puncture is recommended for renal collecting system access, rather than a two-puncture (renal pelvis first, then calyceal) technique
	When anticipating antegrade ureteric stent placement, an interpolar calyceal puncture is recommended
	When placing a nephrostomy catheter, one should avoid traversing the erector spinae muscles as this approach is more painful and associated with higher rates of tube dislodgement
	In the presence of pus or known infection, contrast injection is discouraged as increases in collecting system pressure may provoke bacteraemia and acute uroseptic collapse
	In patients with hyperkalaemia (serum potassium > 6mEq/L) or metabolic acidosis (acidaemia pH < 7.2), the nephrology team should be involved to manage these co-morbidities so as to decrease the risk of intraprocedural arrhythmias or cardioplegia
	All hospitals should have a robust 24/7 management plan for the decompression of infected obstructed kidneys, which may include patient transfer to a neighbouring organisation
	In patients with a short life expectancy (in whom active treatment has been stopped), unilateral metastatic obstruction of the ureter without pain or infection may be left untreated to preserve quality of life
	Maintenance of a stent register is required to ensure appropriate follow-up and exchange for all ureteric stents inserted
	Antegrade ureteric stenting should be avoided in the presence of active urinary tract infection
	The presence of active ipsilateral stone disease is a significant risk factor for stent encrustation, so stent dwell time should be less than 3 months
	It should be ensured that bladder outflow is unimpeded prior to placement of a ureteric stent
	The vesical loop of a ureteric stent should be cranial to the trigone and should not be deployed across the midline, so as to reduce the incidence of stent-related bladder symptoms
	A small volume irritable bladder can predict debilitating stent symptoms with double-J stents and high-volume pelvic malignancy can predict stent failure; in these patients, a trial of stent may be performed using a nephro-ureteric stent, which can be converted to PCN with ease should the patient experience debilitating bladder irritation
	Silicone stents resist extrinsic compression poorly and so should be avoided in cases of malignant extrinsic disease in particular. When economically feasible, metal stents are recommended for the palliative management of malignant ureteric obstruction
	Where possible, ultrasound should be the primary imaging guidance modality for nephrostomy or stent placement in children
	Antegrade ureteric stents should not be placed in the presence of bladder fistulae without additional upper tract diversion via nephrostomy. Diverting nephrostomy may not be required in the presence of ureteral fistulae if they can be managed with covered self-expanding stents

*PCN* Percutaneous nephrostomy

## Methods

The writing group, established by the CIRSE SOP committee, consisted of five clinicians with internationally recognised expertise in this topic and one research assistant (I.I.). The writing group reviewed the existing literature, performing a pragmatic evidence search using PubMed to search for publications in English and relating to human subjects from 2001 to 2025. Relevant older primary sources were included where the data have not been updated.

## Background

Untreated obstructive uropathy is associated with high morbidity leading to renal impairment, end-stage renal failure and death. Particularly when acute, urgent decompression is necessary to prevent compression-mediated ischaemia of the renal parenchyma, the development of irreversible renal failure, and sepsis if the urine is infected.

## Indications

The aetiology of urinary tract obstruction may be malignant (malignant ureteral obstruction (MUO)) or benign (benign ureteric obstruction (BUO)) and may be intrinsic or extrinsic to the ureter (Table 2) [1–3]. Urinary tract obstruction can occur anywhere from the level of collecting system infundibuli to the urethral meatus; however, percutaneous decompression is usually indicated for management of obstruction cranial to, or at, the uretero-vesical junction (UVJ). Percutaneous nephrostomy (PCN) is usually employed when retrograde endourologic approaches have failed or have a poor predicted efficacy due to pre-existing (e.g. renal transplant, urinary diversion) or acute conditions (e.g. obstruction, infection, bleeding).

**Table 2:** Causes of upper urinary tract obstruction

Extrinsic		Intrinsic	
Malignant	Benign	Malignant	Benign
Primary pelvic cancer of GU, gynaecologic or CRC origin	Uterine leiomyomas	Upper tract TCC	PUJ obstruction
	IgG4-related RPF		Ureteric stones
Retroperitoneal lymphadenopathy from primary pelvic cancer	Post-radiotherapy	Mural metastases from carcinoma (breast, lobular)	Soft tissue luminal obstruction (sloughed papillae, fungus ball)
	Idiopathic RPF		
Retroperitoneal infiltration from upper GI malignancy (stomach, pancreas)	Vascular stent graft or vasculitic RPF	Lymphomatous mural infiltration	Mural fibrosis from mycobacterium tuberculosis
Retroperitoneal lymphoma	Iatrogenic injury or ligation of ureter during pelvic surgery		Ureteric strictures

*CRC* Colorectal carcinoma, *GI* Gastrointestinal, *GU* Genitourinary, *IgG* Immunoglobulin G, *PUJ* Pyeloureteric junction, *RPF* retroperitoneal fibrosis, *TCC* Transitional Cell Carcinoma

### ***Indications for Percutaneous Nephrostomy (summarised in ***Table 3***)***

**Table 3:** Indications for percutaneous nephrostomy

Goal of procedure	Example indications	Indications for active management via nephrostomy in pregnancy
Urinary drainage	• Infected renal tract	• Urinary tract infection / sepsis
	• Obstructed renal tract	• Progressive renal impairment
	• Improving renal function to facilitate intravenous chemotherapy	• Unresolved symptoms
		• Failure of conservative treatment
		• Obstruction of a solitary kidney if symptomatic or associated with impairment of renal function
	• Relief of renal colic	
Urinary diversion (non-dilated systems)	• Urinary leak (post-traumatic or iatrogenic) usually in combination with ureteric stenting and drainage of associated urinomas	
Usually temporary and bilateral to allow healing; however, in palliative settings or in patients unfit for reconstruction, diversion may be permanent with the addition of ureteric occlusion with coils, glue, and/or other materials		
	• Urinary fistula (benign and malignant)	
	• Haemorrhagic / post-radiotherapy cystitis	
Tract access for other procedures	• PCNL for stones > 2 cm	
	• Primary ureteric stent placement	
	• Retrieval of stent fragment	
	• Biopsy of renal pelvic/ureteric lesions	
	• Pyeloperfusion during thermal ablation of renal tumours	
	• Ureteric embolisation	
	• Ureteric covered stent placement	
Tract access for tract medication administration	• Instillation of chemotherapy for upper tract TCC	
	• Instillation of antifungal agents in the presence of upper tract infection	

*TCC* Transitional cell carcinoma; *PCNL* Percutaneous nephrolithotomy

1. Urinary drainage for decompression

Urinary drainage is the most common indication, and PCN is particularly indicated in infected obstructed urinary collecting systems (pyonephrosis) to prevent sepsis, loss of renal function, and potentially death. An infected obstructed kidney is an emergency; severe cases are associated with a mortality rate up to 19% if untreated, and delayed decompression is associated with increased mortality [4, 5]. Thus, all institutions should have a robust 24/7 management plan for the decompression of infected obstructed kidneys, which may include patient transfer to a neighbouring organisation. Patients admitted overnight should be medically stabilised and are scheduled for decompression first on the morning list; however, septic patients who do not respond to initial attempts at stabilisation should be decompressed emergently (including overnight), as this will be the life-saving intervention. The presence of a single functioning kidney or a transplant with declining function increases the requirement for emergent decompression. PCN is equally important for the reversal of acute kidney injury associated with non-infected obstructive uropathy.

Hydronephrosis alone does not necessarily indicate drainage, e.g. when caused by pregnancy, diuretics, over-hydration, vesico-ureteric reflux, or megacalycosis. Furthermore, hydronephrosis may persist for quite some time after relief of downstream obstruction, e.g. bladder outflow obstruction. In patients with a short life expectancy (in whom active treatment has been stopped), unilateral metastatic obstruction of the ureter without pain or infection may be left untreated to preserve quality of life by avoiding an external drainage catheter and bag.

2. Urinary diversion

3.Access to the collecting system and ureter for other procedures or procedural adjuncts

4. Access to the collecting system for the instillation of medications

### Specific Clinical Situations

**Pregnancy**

Decision-making for all interventions in pregnancy must balance the risks to the mother with the risks of foetal radiation exposure. Accepted indications for active interventions during pregnancy are included in Table 3.

**Nephrostomy in the presence of renal transplantation**

Indications for nephrostomy in renal transplants (or functional solitary kidneys) are the same as in the non-transplant population; however, the requirement for decompression is more urgent as loss of renal function will not be compensated for by a functioning contralateral kidney.

### Indications for Ureteric Stenting

Radiological antegrade stenting is most often performed as ‘internalisation’ of drainage following acute PCN. Any infection should be cleared before stent insertion. There are four main clinical scenarios for stenting, the indication determining the optimal stent type: temporary, semi-permanent, and permanent (Table 4).

1. *Obstructing, symptomatic ureteric stone*
2. *Malignant Ureteric Obstruction*
3. *Benign Ureteric Obstruction*
4. *Ureteric fistula (if the fistula can be covered with a stent graft)*

**Table 4:** Indications for ureteric stenting

Clinical scenario	Optimal stent choice
1. Obstructing symptomatic ureteric stone	**TEMPORARY**
	Ureteric stent placement prior to definitive ureteroscopy or shockwave lithotripsy facilitates endourologic management in difficult ureteric anatomy, infection, or poor renal function
	Advised in the presence of severe ureteral oedema, anticipated long operative time (> 45 min), and in the presence of renal pelvic or ureteric stones > 2cm [6]
2. Malignant ureteric obstruction	Usually a **PERMANENT** requirement in a palliative setting with the patient’s end-of-life defining disease being their malignancy
	MUO may occasionally resolve with treatment, e.g. lymphoma, in which case the stent can be removed
3. Benign ureteric obstruction	Usually **SEMI-PERMANENT** as the patient’s life expectancy is not related to the ureteric obstruction and stent exchange will be required at some point
	In patients where more definitive surgical reconstruction is not possible; stents with the longest dwell time are preferable but where removal and exchange remain feasible (including combined nephro-ureteric stents)
4. Ureteric fistula (if the fistula can be covered with a stent graft)	**TEMPORARY** until healing, or **SEMI-PERMANENT** if no likelihood of healing

### Indications for Nephro-ureteric Stent Placement

Where repeated access to the urinary tract is needed, e.g. in the management of some ureteric injuries where a combination of collecting system decompression and ureteric splinting is required, including in the renal transplant setting in the management of an anastomotic stricture.

## Contraindications

### **Contraindications to Percutaneous Nephrostomy**

There are no absolute contraindications to PCN. Severe coagulopathy and thrombocytopaenia should be reversed before PCN and, if uncorrectable, would favour retrograde stent insertion if feasible. In the presence of pyonephrosis, critical care management of septic shock should be initiated to stabilise the patient, but drainage of the pus by PCN should not be delayed in the pursuit of the ‘perfect’ patient for intervention. In cases of hyperkalaemia (serum potassium > 6mEq/L) or metabolic acidosis (acidaemia pH < 7.2), nephrology should be consulted to manage these conditions to decrease the risk of dangerous intraprocedural arrhythmias or cardioplegia. In MUO, consideration needs to be given as to whether decompression will lead to improvement in quality, not just quantity, of life.

### Contraindications to Ureteric Stenting

Current active urinary tract infection is a contraindication to ureteric stent insertion. There is no standard time interval between PCN for infected obstructed upper tract and internalisation; however, many practitioners wait at least 3 days from PCN to placement of polymeric double-J stents (DJS) and 7 days for semi-permanent metallic stents. Urinary lithiasis increases the risk of encrustation; therefore, active ipsilateral upper tract stone disease is a ***relative contraindication*** to semi-permanent metal stents and should limit the dwell time for DJS (maximum dwell time should not exceed 2–3 months).

Regardless of cause, stented ureters often lack effective peristalsis and require low vesical pressures for function [7]. Causes of high intra-vesical pressure should be addressed which, in some cases, will obviate the need for stenting, e.g. passage of a urethral catheter in the presence of infravesical obstruction. A small volume, irritable bladder can predict debilitating stent symptoms and high-volume pelvic malignancy can predict stent failure, but these are ***relative contraindications*.** In these patients, a trial of stent may be performed using a nephro-ureteric stent (NUS), which can be converted to PCN with ease should the patient experience debilitating bladder irritation. Similarly, urinary incontinence and neurogenic or spastic bladders are contraindications. Antegrade stents should not be placed in the presence of pelvic fistulae without additional upper tract diversion via PCN.

## Patient Preparation

Informed consent must be obtained, preferably by a member of the IR team and where possible at a suitable interval before the procedure, e.g. 2–3 days beforehand in the Interventional Radiology outpatient clinic [8]. In the presence of lower tract fistulae, multidisciplinary discussion is recommended to ensure the optimal management.

### Pre-procedural Imaging

Adequate pre-procedural imaging is required for diagnosis and procedure planning. Hydronephrosis is clearly demonstrated by ultrasound. Computed tomography of the renal tract is optimal for demonstrating calculi (conventional CT for radiodense calculi, dual-energy CT for radiolucent stones) [9, 10]. Excretory contrast-enhanced CT (CECT) demonstrates the site and cause of obstruction in non-calculous disease and documents urinary leakage/fistulation. MR-urography can provide similar diagnostic information and can identify radiolucent stones, but availability may be challenging in some departments, especially out of hours. Imaging should be reviewed specifically to optimise the approach, particularly with regard to the presence of possible anatomic variants (e.g.horseshoe kidney) and to avoid puncturing vessels or bowel. In elective cases, radionuclide scintigrams may contribute to the decision-making process for the procedure, particularly in differentiating true from pseudo-obstruction, in assessing residual function in a kidney prior to proposed interventions, and in prioritising which side to drain in cases of bilateral obstruction.

### Diagnosing Stone Disease in Pregnancy

Ultrasound (US) is the first-line examination. If inconclusive, non-contrast MRI may be used as a problem-solving tool in the first trimester [11]. Low-dose CT (LDCT) can be used in the second and third trimesters for complex cases [11, 12].

### Laboratory Work-up

PCN carries a moderate to high risk of bleeding [13]. Coagulation status needs to be evaluated and corrected when disturbed. The following are generally taken as acceptable parameters: haemoglobin > 8g/L, platelets > 50 × 10 ^9^/L, and prothrombin time ≤ 15s (INR ~ 1.5). Blood urea nitrogen, creatinine, haematocrit, and urinalysis are routinely checked, with urine cultures reserved for cases of suspected infection.

### General Preparation Including Antibiotic Administration

Local institutional pre-procedure fasting requirements should be followed, hydration maintained according to the patient’s clinical status, and peripheral venous access obtained. Patients presenting with sepsis secondary to an infected obstructed collecting system should be treated with empiric broad-spectrum antibiotics until the responsible pathogen is identified on culture of urine aspirated at the time of nephrostomy [14]. Routine prophylactic administration of pre-procedural antibiotics in patients without evidence of infection is a point of debate without clear supporting evidence. Local protocols, as defined by microbiology and pharmacy, should be followed. Where appropriate, prophylactic antibiotics are administered 1 h before the procedure and are usually not continued if there is no evidence of infection at the time of puncture, unless recognised risk factors are present. For retrograde transurethral exchange of ureteric stents, a single dose of a third-generation cephalosporin is adequate.

## Treatment and Peri-procedural Care

### Common to all Procedures

#### Intraprocedural Monitoring and Medications

Pulse, blood pressure, and oxygen saturations must be monitored during the procedure. If sedation is given, cardiac monitoring is recommended. The urothelium may be sensitive, and wire and/or catheter manipulations can provoke a bradyarrhythmia. Nephrostomy can be performed with local anaesthesia only, the latter administered to the skin, the subcutaneous tract, and renal capsule. In stable patients, intravenous sedation can be used as required.

### Nephrostomy for Decompression

#### Positioning and Tract Planning

Patients are placed in a prone, semi-prone, or decubitus position. Immediately pre-procedure, targeted US is performed to identify the optimal patient position and skin entry site and to plan a tract that excludes interposed organs, bowel, or vasculature. A posterolateral approach to the most suitable calyx is recommended so as to puncture the parenchyma via the relatively hypovascular plane of Brödel, which usually corresponds to the posterior axillary line. One should avoid traversing the erector spinae muscles as this approach is more painful and associated with higher rates of tube dislodgement. If the PCN is going to be permanent, and a more anterior approach is safe, this is more comfortable for the patient when sleeping and facilitates bag exchange.

When anticipating antegrade stent placement, an interpolar approach should be considered to avoid the mechanical disadvantage arising when a lower pole calyx is used, particularly in the presence of a tight ureteric stricture. Although a subcostal approach is preferred, intercostal approaches (cranial to the 12th or rarely the 11th ribs) may be necessary, especially when targeting upper pole calculi for percutaneous nephrolithotomy (PCNL). When employed, these approaches are associated with higher risks of pleural puncture [15].

### Procedural Technique

Under sterile conditions, the target calyx is punctured under ultrasound guidance with a needle of the operator’s choice, more experienced operators using 16–19-gauge needles, more recently trained IRs using a 21-gauge two-part needle. A standard Seldinger technique is most frequently employed in adults, often utilising a proprietary triaxial set to upsize from the 0.018″ nitinol-tipped guide wire used for initial entry to a 0.035 or 0.038″ stiff guidewire. In the presence of a scarred cortex, initial access with 16–19G needles facilitates direct passage of a 0.035″ wire which will provide more mechanical advantage when dilating the tract. In fibrous or scarred cortices, it may be necessary to dilate to 1 French larger than the planned catheter. In the presence of pus or known infection, contrast injection is discouraged as increases in collecting system pressure may provoke bacteraemia and acute uroseptic collapse. For the same reason, wire manipulations other than those required to safely place the catheter should be avoided. Following tract dilatation, the catheter is inserted over the wire and its loop formed within the collecting system. The same technique is used in ***obstructed transplant kidneys*** but is performed with the patient in the supine position.

Means of securing the catheter to skin vary widely and are at the operator’s discretion; however, suturing the catheter to skin is discouraged as it is painful for the patient post-procedure and has a risk of suture-tract infection. An open system (nephrostomy draining into a stoma bag) has a lower rate of nephrostomy displacement.

### Nephrostomy for Urinary Diversion

Calyceal access can be challenging in the absence of distension. The procedure should be attempted using US guidance initially, aiming for the central apex of the renal pyramid in the mid or lower pole [16], subjacent to which the target calyx is expected, using Doppler to identify and avoid vasculature. Even when calyceal puncture is successful, spontaneous return of urine is not anticipated as the collecting system is not under pressure and the path of least resistance for urine remains via the ureter rather than the 21G needle. Gentle injection of contrast under fluoroscopy is recommended to confirm intra-calyceal location. Once confirmed, a 0.018″ wire is advanced under fluoroscopic guidance into the renal pelvis and access upsized as above [17].

In cases where access is not easily obtained, the administration of intravenous fluid and contrast in combination with a diuretic agent resulting in pelvicalyceal opacification and mild distension can facilitate subsequent US and fluoroscopic-guided puncture [18]. Where available, intravenous injection of contrast followed by cone beam CT with proprietary tracking software can assist in targeting a suitable calyx [19–21]. In obese patients or patients with complex clinical scenarios, e.g. critically ill or recently operated, as well as in patients with non-dilated collecting systems, CT-guided nephrostomy has been shown to be an acceptable alternative [22, 23]. If these approaches fail, a combined approach with urology may be necessary.

### Nephrostomy for Percutaneous Nephrolithotomy Access

US-guided access is recommended in addition to fluoroscopy as it allows one to identify non-target anatomy (colon, spleen, and liver) and to maximise the length of the parenchymal tract, which contributes to secure positioning of the sheath. If ureteric occlusion catheters have been placed cystoscopically pre-procedure, the collecting system can be temporarily distended via retrograde infusion.

### Ureteric Stent Insertion

Ureteral stent insertion can be performed in combination with primary nephrostomy placement in one sitting, or as a two-stage procedure (initial nephrostomy with second stage stenting once dilatation has resolved and infection is excluded/resolved). In chronic obstruction, the ureter may be very dilated and tortuous at initial nephrostomy, rendering access to the bladder from above difficult. If stenting is not possible at the first attempt, it is recommended to allow 48–72 h of nephrostomy decompression, permitting recovery of mucosal oedema and reduction in tortuosity, following which a further attempt to traverse the ureter is usually successful.

### Procedural Technique

If performed in the same sitting as initial access, a 5 Fr introducer sheath is advanced to the renal pelvis and standard techniques are used to direct a hydrophilic wire and catheter combination past the level of obstruction to the bladder. If resistance is encountered, ureterography is performed to identify the level of obstruction and characterise the morphology of the lesion. Traversing exceptionally tight ureteric strictures may require additional proximal support with a longer vascular sheath advanced into the ureter and the use of a microwire/microcatheter combination to cross the stricture. Ureteroplasty with a 6–8 mm angioplasty balloon may be required prior to stent placement in such cases.

Before the wire is advanced into the bladder, the patient should be warned that they will experience transitory genito-perineal pain. If there is difficulty in advancing a wire across the UVJ, ensuring that the bladder is distended can alter the angle of insertion at the UVJ, facilitating crossing of the stricture [24]. Bladder access should be confirmed with the injection of contrast. Additional distension may be required to ensure adequate space for the caudal loop of the stent to form (urethral catheters should be clamped beforehand). The hydrophilic wire should be exchanged for a stiff working wire. In cases of ureteric transection, a rendezvous approach with an urologist working from below may be required, facilitating subsequent stent deployment [25–27]. In these cases, healing will be supported by leaving a proximal diversion nephrostomy catheter in situ after stenting [28]. Additionally, to promote ureteric healing in cases of transection, secondary urinomas should also be drained, with placement of the catheter close to the level of leakage [29].

The introducer sheath in the renal pelvis is then exchanged for a peelaway sheath following tract dilatation, and the stent advanced over the wire to the bladder. When the stent has been advanced into the bladder, the wire is withdrawn, allowing the caudal loop to form; the optimal final position being cranial to the trigone in the ipsilateral half of the bladder, i.e. not crossing the midline, to avoid stent-related symptoms [30, 31]. The wire is then withdrawn further, and the cranial loop formed within the renal pelvis.

Following ureteric stent placement, the requirement for a ‘covering’ nephrostomy is decided on a case-by-case basis, the most common indication being a traumatic procedure with extensive clot in the collecting system at procedure completion (a matter of debate as the risk of stent occlusion is reduced by the intrinsic lytic effect of urine). If there are concerns regarding early stent patency, e.g. a plastic stent in a tightly compressed ureter, it is deemed prudent to leave a nephrostomy in situ to facilitate nephrostography 48–72 h post-stent insertion. When using wide-bore metal stents, if patency can be confidently confirmed at the time of placement with contrast injection, there is no clear indication for a covering nephrostomy [32].

In cases where maintenance of renal access is preferred (e.g. documented rapid stent encrustation), combined one-piece NUS are useful tools as they can be capped externally when patent without the need for a drainage bag; however, when they start to occlude, they can be exchanged via the original access. This approach also allows IR to take ownership of exchanges without the need for transurethral exchanges by the urology team.

### Advantages and Disadvantages of the Different Types of Ureteric Stent

The choice of stent must be matched to the clinical scenario, local availability, and expertise.

#### Ureteric Stent Types

**Polymeric**

The classic “Double-J” (DJS) or “Double Pigtail” stent is usually made of polyurethane plastic with a variety of coatings to reduce encrustation and bacterial biofilm and improve patency. Sideholes may be either along the length of the stent or just in the terminal pigtails. These are available in differing lengths, chosen for each individual patient on the basis of their height [33] or the measured distance from the pyeloureteric junction (PUJ) to the UVJ (typically between 20 and 30cm, best estimated on CT reconstructions) [33–36]. These stents are loaded on a removable central stiffener with a variety of releasing mechanisms and come in diameters from 4 to 10 Fr. Suggested dwell times prior to planned exchange vary from 3 to 12 months; the individual manufacturers’ instructions for use should be consulted.

**Metal “double-J”**

A chromium–nickel alloy in a spiral structure that allows ingress of urine at every point above and egress at every point below the stricture. It is 5–6 times the unit cost of a polymeric stent but is licenced for 12 months indwell, comes in a standard 6 Fr diameter size, may extend from 20 to 30cm in length, and is intended to cover the length of the ureter.

### Self-expanding metal ureteric stents (SEMS)

Unit costs are up to 20 times that of a polymeric stent; however, their dwell times are over 12 months, reducing the costs associated with multiple exchanges.

### Stent selection in MUO

The average life expectancy of patients with MUO (median survival 210 days) is longer than the dwell time of polymeric stents, but shorter than that of metal stents [37]; thus, primary metal stenting permits an almost “stent and forget” approach, leading to increased quality of life and no stent exchange requirement (unless the patient survives to 1 year post-insertion, at which time exchange is recommended [38]).

### Ureteric obstruction and Stone Disease in Pregnancy

The use of stents is discouraged in pregnancy due to the increased encrustation rate. Where possible, PCN placement is preferred before the 22nd week [39].

### Ureteric Stent Exchange

When long-term stenting of benign or malignant ureteric strictures is required, scheduled routine exchange is recommended every 4–6 months as polymeric stents are prone to occlusion. The fluoroscopic approach has been shown to be a reasonable alternative to cystoscopy, especially in women [40, 41], albeit coming with an increased radiation burden and increased procedure time. In men, the procedure is more challenging due to the longer urethra and, if the stent is withdrawn too caudally, the cranial loop may be delivered entirely into the bladder, losing access to the ureter [41, 42].

### Fluoroscopic Exchange Technique

All patients should receive a single prophylactic dose of a broad-spectrum intravenous antibiotic pre-procedure, typically a third-generation cephalosporin. The procedure is performed under conscious sedo-analgesia and topical Xylocaine gel. Local asepsis with Chlorhexidine-based, rather than Betadine-based agents, is recommended as the latter are irritating, particularly to female genitals.

Via a urinary catheter, the bladder is distended with roughly 200 cc of dilute contrast, and the catheter is exchanged over an 0.035″ wire for a vascular introducer sheath (at least 2 French larger than the stent to be removed); the sheath limits the urethral irritation that occurs with snare manipulation. Pain from attempted stent removal may be so debilitating as to result in procedure abandonment [43].

The caudal pigtail of the stent is grasped with a snare under fluoroscopic guidance and delivered to the urethral orifice via the sheath. Once delivered, the caudal endhole of the stent is cannulated with a wire which is advanced and coiled within the renal pelvis. The stent is then removed and replaced over the wire. Essentially, the same approach is taken when exchanging stents in the renal transplant and trans-ileal conduit settings [44, 45]. In the presence of a conduit, if instead of a stent, a pigtail catheter is used with its hub delivered into a stoma bag, exchange is much more straightforward.

Severely encrusted stents may result in an inability to advance a wire through the stent. In these cases, the length of the stent may be ‘extended’ by passing a suture through the caudal tubing and advancing a long sheath over the suture–stent combination into the ureter, facilitating stent removal whilst maintaining access to the ureter. A similar approach is taken in the unusual event of needing to exchange a metallic stent, whereby, on delivery to the meatus, a suture can be tied tightly around the stent and a long sheath advanced over the suture–stent combination into the ureter.

### Specific Considerations for Paediatric Patients

Almost all nephrostomy and stent placements in children will require sedation or anaesthesia, the selection depending on established practices and local expertise. It is advised that these procedures be performed in specialised centres with paediatric urology back-up in the event of complications. Children require specialised sedation and anaesthesia services tailored to their age and weight. A multidisciplinary approach involving paediatric specialists (nephrologists, urologists, anaesthesiologists, nurses, and others) is recommended. Children requiring renal or ureteric interventions range from less than 1kg to 100kg and require the use of a variety of needles, wires, catheters, and drainage devices tailored to their anatomy and size.

Minimising radiation exposure is particularly important. Where possible, US should be the primary modality during the procedure and where this is not possible, the use of low-dose pulsed fluoroscopy with last image-hold is highly desirable. A high-quality US system with a variety of transducers is essential to permit imaging across a range of patient sizes and weights.

The practical steps of PCN and stent placement in school-age children are very similar to those in adults, though kidneys tend to lie more cranial than in adults and upper pole punctures are often not possible; however, nephrostomy placement in neonates or infants presents challenges not generally encountered in adults. Neonatal kidneys are small (average 4.5cm). Despite their relative proximity to skin, the lack of retroperitoneal fat makes the kidneys mobile and liable to retreat from an advancing needle or trocar. Even though the collecting system may be relatively dilated, its absolute volume is small, so coiling guidewires and forming a pigtail may be challenging. If the guidewire does not advance down the ureter, it can be impossible to get the stiff portion of the guidewire beyond the renal capsule. The small renal pelvic volumes also mean the collecting system can decompress rapidly during the procedure. The combination of these factors may make it difficult to advance a micropuncture set into a collecting system.

The approach used (21G single needle access versus modified trocar versus direct trocar) will be informed by the clinical experience of the operator, combined with patient factors such as the degree of pelvicalyceal dilatation. Koral et al. advocated a modified trocar placement approach [46] with a 19-gauge vascular single-wall needle through which a 0.035″ 80-cm stiff guidewire is passed. Under fluoroscopy, a 6-French hydrophilic tapered tip catheter and small locking pigtail are advanced over the wire. Other authors have suggested a direct trocar approach for neonates, in particular those with Grades III or IV hydronephrosis [47]; this approach requires excellent visualisation of the calyceal trajectory and may be a technique better selected for smaller children [48].

In addition to the indications in adults, stent placement in children is indicated for persistent PUJ obstruction following pyeloplasty and UVJ stenosis following reimplantation. Antegrade stent placement is frequently possible when retrograde cystoscopic attempts have failed. Internal stents range in size from 4–8 French and are available in variable lengths (typically 8–20cm stent length or 22-32cm length) which are invaluable for paediatric practice. A useful heuristic for estimating the length of a child’s ureter is ‘age in years + 10cm’. NUS in children are prone to infection and dislodgement by pulling on the external component and thus internalised drainage is preferred. Metallic stents are never used, even in the presence of MUO. Nephrostomy catheter dislodgement and catheter-associated urinary tract infections are common post-procedural complications and need to be actively sought and managed [49].

## Post-treatment and Follow-up Care

Post-procedure, patients require close monitoring in order to detect early uroseptic collapse or evidence of haemorrhage. If the patient was profoundly septic pre-procedure, High Dependency Unit (HDU) or Intensive Care Unit (ICU) monitoring is recommended post-procedure. The patient’s fluid balance should be monitored closely as post-obstructive diuresis may occur. Antibiotics are continued in the presence of ongoing infection in the post-procedure period, adjusted according to urine cultures. Post-procedural pain is managed in line with hospital protocols. The output from the nephrostomy should be monitored by volume and to detect onset, or worsening, of haematuria. Following stent placement, the patient should be warned that they will likely experience some urinary frequency and bladder irritation in the first 48–72 h but that this should resolve.

## Clinical Follow-up: Nephrostomy Catheters

When the underlying cause of ureteric occlusion or urinary leakage is resolved, the nephrostomy catheter can be removed. If there is no ureteric stent in situ, removal can be safely performed without fluoroscopic guidance once the locking mechanism has been released; however, if a ureteric stent is present, the nephrostomy should be removed over a wire with fluoroscopic guidance.

If there is an ongoing requirement for drainage, e.g. in malignancy, nephrostomy catheters should be routinely exchanged every 6–8 weeks initially, as they tend to occlude secondary to encrustation after some time, with the attendant risk of sepsis. If, on repeat visits, the patient’s catheter is not encrusted, the length between exchanges may be safely extended (8–12 weeks). Flushing of the catheter with saline twice a week may reduce the risk of occlusion. In addition, patients should be advised to maintain a moderately high oral fluid intake, e.g. 2L/day.

In pregnancy, nephrostomy catheters should be changed more frequently, i.e. every 4–6 weeks, especially if the patient is a stone-former. If signs or symptoms of tube obstruction/malposition occur, such as fever, flank pain, or pericatheter leakage, the catheter should be exchanged on an urgent basis (within 24 h), regardless of the planned exchange schedule.

All patients should be provided with 24-h contact information and should be instructed to contact the hospital if they develop fevers, flank pain, or the catheter dislodges or stops draining, or urine leaks alongside the catheter. If a ‘trial of capping’ is being conducted as an outpatient, the patient should be given drainage bags and advised to uncap the tube and connect it to a drainage bag if they develop any of the symptoms above.

## Clinical Follow-up: Ureteric Stents

All patients receiving a stent should be entered into a hospital stent register with a clear plan of management (e.g. planned routine exchange 4–6 months). This is especially true for patients cared for by non-urology specialities, e.g. Gynaecology and Oncology, in order to reduce the incidence of ‘forgotten stents’ [50]. Urinary lithiasis increases the risk of encrustation; therefore, active ipsilateral upper tract stone disease should limit dwell time to less than 3 months. All stents are prone to failure at some point, with recurrence of acute kidney injury, obstructive pyonephrosis, or intractable stent symptoms from ureteric dilatation or trigonal irritation, and patients should be reviewed specifically for these issues. In **pregnancy**, stents migrate more frequently because of the physiological hydroureter and have a high risk of encrustation and bacterial colonisation; for these reasons, the use of stents in pregnancy is discouraged [51].

**The complications** of nephrostomy are summarised in Table 5.

**Table 5:** Complications of nephrostomy insertion

1	Non-target organ puncture (pleura or gut)	Occurs in between 0.1 and 0.2% of PCNs [52–58]
		Pleural complications are more frequent in supracostal approaches and occur more often on the right (29%) than the left (19%), and in 11th intercostal space punctures [15]
2	Bleeding	Transfusion required in 1.9–4% [17, 54, 55, 59, 61–64] the need increasing in coagulopathic patients [59]
		Incidence of haematuria is increased when **PCNL is performed** (12–14%) [65] and when puncturing **non-dilated** systems (31.5%) [17]
		Vascular injury requiring intervention such as embolisation or nephrectomy occurs in between 0.1 to 1% [54, 61, 63]
3	Infection	In the non-emergent setting, bacteraemia occurs in 21% of patients
		Low-grade fevers occur in as many as 100% following nephrostomy in the emergency setting [65]
		In the presence of pyonephrosis, septic shock occurs at the time of nephrostomy in 7–9% of patients [53–55, 59, 61, 64, 67, 68] and in 3.6% will lead to an ICU admission [64]
4	Catheter malfunction (blockage / inadvertent dislodgement)	Dislodgement occurs < 1% in the early post-procedure period [59, 64]
		Catheter dislodgement occurs in the first month after insertion in between 3.6% and 5% of PCNs in native kidneys, increasing to as many as 62% by 36 months [66, 70]
		Nephrostomy tube occlusion requiring exchange occurs reasonably often (1.73%), incidence increasing with dwell time [66]

*PCN* Percutaneous nephrostomy; *PCNL* Percutaneous nephrolithotomy; *ICU* Intensive care unit

**Non-target organ puncture** occurs infrequently (0.1 and 0.2% of PCNs) [52–58]. Supracostal approaches are associated with a higher risk of pneumo-hydrothorax (especially during PCNL), empyema, haemothorax, and nephro-pleural fistula. Key to managing these complications in the post-procedural period is to exclude downstream obstruction by ureteric stent placement.

**Bleeding** typically occurs due to branch lacerations [59] and is more common in infundibular and direct renal pelvic punctures [54, 60]. In non-dilated systems, there is a sixfold increase in overall complications compared to puncture of a dilated system [54]. Persistent severe haematuria lasting more than 3 days should prompt suspicion of an arterial injury and should be investigated by CT angiography in the first instance. If the latter does not reveal a source, combined renal angiography and nephrostomy removal over a wire should be performed to identify and treat the bleeding vessel via the transarterial approach.

**Intraprocedural death** has not been reported; however, deaths have occurred in the first 30 days post-procedure, occurring in 0.192% in Kaskarelis et al.’s series [66].

**Infection:** In the presence of pyonephrosis, septic shock occurs at the time of nephrostomy in 7–9% of patients [53–55, 59, 61, 64, 67, 68] and in 3.6% will lead to an ICU admission [64].

**Catheter malfunction (blockage/inadvertent dislodgement):** The ability to reinsert a new catheter via the prior tract increases with age/maturation of the tract, being close to 100% of tracts > 12 months old. A need for frequent tube changes, particularly if associated with pain and/or irritative GU tract symptoms, can result in a diminished performance status and reduced quality of life [31, 69].

**The complications** of ureteric stent placement and exchange are summarised in Table 6.

**Table 6:** Complications of ureteric stent placement and exchange

Complication	Description	Frequency
Wire perforation of the ureter	Extravasation may occur when crossing very tight strictures; rarely significant if a diverting nephrostomy is also placed [24]	
Maldeployment	Stents deployed without addressing the level of obstruction, e.g. distal pigtail above UVJ or stricture not fully covered	
	Rarely, maldeployment into the retroperitoneum may occur if mural perforation is not recognised	Rare, occurring in 0.3% of patients in one large 25-year review [71]
Stent-related symptoms	Urge incontinence, haematuria and pain	Affects 80–90% of patients [72, 73]
Macroscopic haematuria	Severe persistent haematuria, particularly if presenting late, should raise suspicion for rare uretero-arterial fistulation (more common in elderly patients with a combination of pelvic surgery and pelvic radiotherapy) [38]	In 1 to 7%, requiring blood transfusion in only 0.0014% [38, 71]
Crystal incrustation / encrustation	Common in the presence of polymeric stents (less often on metal stents); when severe, encrustation may lead to stent fracture at the time of attempted exchange [24, 71, 74–76]	Increases in proportion to dwell time, occurring in up to 76.3% of patients after 12 weeks
‘Forgotten’ ureteric stents	Ureteric stents may become severely encrusted and serve as a nidus for stone formation and infection, rarely resulting in a need for nephrectomy [71, 77, 78]. In some instances, if a stent is ‘stuck’ it may be possible to pass a new stent alongside the fixed stent to allow drainage	
Infected stents	May occur leading to pyuria, generally prevented by routine exchanges (every 3–6 months) [31, 73]	
Stent migration	Stents may migrate or become displaced	3.57% [66]
Strictures	Strictures may develop secondary to a longstanding stent, particularly when encrusted, which can hinder or prevent subsequent exchange [43]	
Complications: stent exchange	Transient (< 1 day), self-limiting minor haematuria	Overall complication rate 5%
	Urinary tract infection most common	7.7%
	Procedure failure (Grade 1b CIRSE) due to:	4.9%
	- Failure to grasp the caudal end of the stent	1.8%
	- Procedure discontinuation secondary to patient discomfort	1.1%
		[41, 43]

*UVJ* Uretero-vesical junction

## Outcomes

Percutaneous nephrostomy and ureteric stent placement have high technical and clinical success rates, summarised in Tables 7, 8 and 9.

**Table 7:** Technical and clinical success rates in PCN

Clinical scenario	Technical Success rates	Clinical Success
Obstructed renal tract	82–99% [54, 63]	Renal function expected to recover in 60% of patients within 2 weeks [79]
Pyonephrosis	98% (54)	PCN has a low procedure-related morbidity (14%) and mortality (2%) [67]
		Inflammatory markers and pyrexia respond more promptly following decompression via PCN compared to retrograde stenting [80, 81]
		Fever and pain improve within 1 – 2 days [82], recovery improved by culture-specific antibiotics (PCN cultures positive in 58% in comparison to 30% of bladder urine cultures) [67]
		Infected, obstructed kidneys may have mortality rates as high as 19%, delayed decompression is associated with increased mortality [4, 5]
Non-dilated systems	82–96% [17, 23]	
Complex stone disease [2], e.g. staghorn calculi	82–85%	
Renal transplants	98–100% (higher success rates due to proximity to skin [83])	
Haemorrhagic cystitis		71% resolution at a median of 12 days post-procedure [84]

*PCN* Percutaneous nephrostomy

**Table 8:** Outcomes in ureteric stenting and exchange

Procedure	Technical success rates	Clinical success	Notes
Primary stenting without covering nephrostomy	88% technical success (23)	83% clinical success [32]	
Secondary ureteric stenting	Close to 100% technical success (23)	98% clinical success [32]	
Fluoroscopic retrograde ureteric stent exchange	Technical success 86–97.5% in females (excluding cystoscopic-assistance) [40, 41, 43, 85, 86]; success reduced with longer intervals between exchanges and in bilateral exchange	Clinical Success: 95.7% (lower in urological / gynaecological tumours (compared to fibrosis / other causes) and in bilateral stent exchange) [41]	
Malignant ureteric obstruction		Metallic stent patency:	Metal stents more cost effective than polymeric [37, 87]
		• 86% at 6 months	
		• 60% at 1 year	
Ileal conduit	Close to 100%	High success rates reported in the management of hydronephrosis secondary to ureteric strictures using a combination approach of initial PCN placement followed by nephro-ureteral stent placement, with subsequent internalisation and recurring retrograde stent exchange via the stoma [88, 89]	
Stone disease		Ureteric stenting, prior to definitive treatment (URS or shockwave lithotripsy), shortens subsequent theatre time and increases SFR in renal calculi [6] with a cost of a longer operative time [49]	
		Unplanned post-procedural hospitalisation in high-risk stented patients is lower than in unstented patients [49]	
		*Post-ureteroscopy* stenting reduces ureteroscopy-related strictures, caused by stone-related trauma, the intervention, or the combination	

*MUO* malignant ureteric obstruction *URS* ureteroscopy, *SFR* stone-free rates, *PCN* Percutaneous Nephrostomy

**Table 9:** Factors predicting ureteric stent failure

Factor	Notes
Bilateral obstruction	• A marker of advanced disease and the most consistent predictor of failure which occurs in 85% of patients with bilateral obstruction compared to 25% with unilateral [38]
Others:	
• Primary malignancy *not* of Gynae, GI or GU origin	
• Serum creatinine > 1.2 mg/ml	
• Patient on no active treatment for primary malignancy	
• Presence of a uretero-conduit anastomosis	
Metallic vs polymer stent	• Polymeric have lesser patency rates compared to metallic (1-year patency rate; 61.1 vs 78.4%) [38]
	• No difference between the stents in the rates of stent occlusion due to abdominal dissemination, lymph node metastasis or direct compression by tumour [38]

*Gynae* Gynaecological; *GI* Gastrointestinal; *GU* Genitourinary

## Conclusions

Percutaneous nephrostomy and ureteric stent placement are frequently performed, minimally invasive procedures which, when performed with appropriate indications and technique, are safe and clinically effective. The writing group’s recommendations are summarised in Table 1.

## Abbreviations

BUO: Benign ureteral obstruction
CECT: Contrast-enhanced CT
CRC: Colorectal carcinoma
DJS: Double J stent
GI: Gastrointestinal
GU: Genitourinary
Gynae: Gynaecological
HDU: High dependency unit
ICU: Intensive care unit
IgG: Immunoglobulin G
INR: International normalised ratio
IVU: Intravenous urography
LDCT: Low-dose CT
MUO: Malignant ureteral obstruction
NUS: Nephro-ureteric stent
PCN: Percutaneous nephrostomy
PCNL: Percutaneous nephrolithotomy
PUJ: Pyeloureteric junction
RPF: Retroperitoneal fibrosis
SOP: Standards of Practice
SFR: Stone-free rates
TCC: Transitional cell carcinoma
URS: Ureteroscopy
US: Ultrasound
UVJ: Uretero-vesical junction

## References

[1] Izumi K, Mizokami A, Maeda Y, Koh E, Namiki M. Current outcome of patients with ureteral stents for the management of malignant ureteral obstruction. J Urol. 2011;185(2):556–61. 21168872 10.1016/j.juro.2010.09.102

[2] Izumi K, Shima T, Shigehara K, Sawada K, Naito R, Kato Y, et al. A novel risk classification score for malignant ureteral obstruction: a multicenter prospective validation study. Sci Rep. 2021;11(1):4455. 33627826 10.1038/s41598-021-84054-7 PMC7904864

[3] Hsu L, Li H, Pucheril D, Hansen M, Littleton R, Peabody J, et al. Use of percutaneous nephrostomy and ureteral stenting in management of ureteral obstruction. World J Nephrol. 2016;5(2):172–81. 26981442 10.5527/wjn.v5.i2.172 PMC4777789

[4] Haas CR, Li G, Hyams ES, Shah O. Delayed decompression of obstructing stones with urinary tract infection is associated with increased odds of death. J Urol. 2020;204(6):1256–62. 32501124 10.1097/JU.0000000000001182

[5] Borofsky MS, Walter D, Shah O, Goldfarb DS, Mues AC, Makarov DV. Surgical decompression is associated with decreased mortality in patients with sepsis and ureteral calculi. J Urol. 2013;189(3):946–51. 23017519 10.1016/j.juro.2012.09.088

[6] Lumma PP, Schneider P, Strauss A, Plothe KD, Thelen P, Ringert RH, et al. Impact of ureteral stenting prior to ureterorenoscopy on stone-free rates and complications. World J Urol. 2013;31(4):855–9. 22037634 10.1007/s00345-011-0789-6 PMC3732763

[7] Rackson ME, Mitty HA, Dan SJ, Train JS. Elevated bladder pressure: a cause of apparent ureteral stent failure. AJR Am J Roentgenol. 1988;151(2):335–6. 3260724 10.2214/ajr.151.2.335

[8] Cazzato RL, de Rubeis G, de Marini P, Auloge P, Dalili D, Weiss J, et al. Interventional radiology outpatient clinics (IROC): clinical impact and patient satisfaction. Cardiovasc Intervent Radiol. 2021;44(1):118–26. 33089359 10.1007/s00270-020-02677-1

[9] Ascenti G, Siragusa C, Racchiusa S, Ielo I, Privitera G, Midili F, et al. Stone-targeted dual-energy CT: a new diagnostic approach to urinary calculosis. AJR Am J Roentgenol. 2010;195(4):953–8. 20858824 10.2214/AJR.09.3635

[10] Cheriachan D, Arianayagam M, Rashid P. Symptomatic urinary stone disease in pregnancy. Aust N Z J Obstet Gynaecol. 2008;48(1):34–9. 18275569 10.1111/j.1479-828X.2007.00798.x

[11] Fulgham PF, Assimos DG, Pearle MS, Preminger GM. Clinical effectiveness protocols for imaging in the management of ureteral calculous disease: AUA technology assessment. J Urol. 2013;189(4):1203–13. 23085059 10.1016/j.juro.2012.10.031

[12] Lewis DF, Robichaux AG, Jaekle RK, Marcum NG, Stedman CM. Urolithiasis in pregnancy Diagnosis, management and pregnancy outcome. J Reprod Med. 2003;48(1):28–32. 12611091

[13] Hadi M, Walker C, Desborough M, Basile A, Tsetis D, Hunt B, et al. CIRSE standards of practice on peri-operative anticoagulation management during interventional radiology procedures. Cardiovasc Intervent Radiol. 2021;44(4):523–36. 33474606 10.1007/s00270-020-02763-4

[14] Chehab MA, Thakor AS, Tulin-Silver S, Connolly BL, Cahill AM, Ward TJ, et al. Adult and pediatric antibiotic prophylaxis during vascular and IR procedures: a society of interventional radiology practice parameter update endorsed by the cardiovascular and interventional radiological society of Europe and the Canadian association for interventional radiology. J Vasc Interv Radiol. 2018;29(11):1483-501 e2. 30274857 10.1016/j.jvir.2018.06.007

[15] Hopper KD, Yakes WF. The posterior intercostal approach for percutaneous renal procedures: risk of puncturing the lung, spleen, and liver as determined by CT. AJR Am J Roentgenol. 1990;154(1):115–7. 2104692 10.2214/ajr.154.1.2104692

[16] Lin F, Yu W, Rao T, Ning J, Ruan Y, Xia Y, et al. The anatomic structure of a fused renal pyramid and its clinical significance in the establishment of percutaneous renal access. Urology. 2019;124:38–45. 30445123 10.1016/j.urology.2018.11.004

[17] Ho Won J, Jin Yang W, Hoon Shin J, Woo Kim J, Ho Chu H, Min Lee S, et al. Percutaneous nephrostomy for nondilated renal collecting system with ultrasound and fluoroscopic guidance: the results of a 10-year experience. Diagn Interv Radiol. 2022;28(3):244–8. 35748207 10.5152/dir.2022.20728 PMC9634922

[18] Yagci C, Ustuner E, Atman ED, Baltaci S, Uzun C, Akyar S. Diuretic agent and normal saline infusion technique for ultrasound-guided percutaneous nephrostomies in nondilated pelvicaliceal systems. Cardiovasc Intervent Radiol. 2013;36(2):492–7. 22893420 10.1007/s00270-012-0461-6

[19] Ritter M, Rassweiler MC, Hacker A, Michel MS. Laser-guided percutaneous kidney access with the Uro Dyna-CT: first experience of three-dimensional puncture planning with an ex vivo model. World J Urol. 2013;31(5):1147–51. 22391646 10.1007/s00345-012-0847-8

[20] Vicentini FC, Botelho LAA, Braz JLM, Almeida ES, Hisano M. Use of the Uro Dyna-CT in endourology - the new frontier. Int Braz J Urol. 2017;43(4):762–5. 28338302 10.1590/S1677-5538.IBJU.2016.0413 PMC5557454

[21] Jiao D, Zhang Z, Sun Z, Wang Y, Han X. Percutaneous nephrolithotripsy: C-arm CT with 3D virtual navigation in non-dilated renal collecting systems. Diagn Interv Radiol. 2018;24(1):17–22. 29256866 10.5152/dir.2017.17079 PMC5765923

[22] Brandt MP, Lehnert T, Czilwik T, Borgmann H, Gruber-Rouh T, Thalhammer A, et al. CT-guided nephrostomy–an expedient tool for complex clinical scenarios. Eur J Radiol. 2019;110:142–7. 30599852 10.1016/j.ejrad.2018.11.028

[23] Sommer CM, Huber J, Radeleff BA, Hosch W, Stampfl U, Loenard BM, et al. Combined CT- and fluoroscopy-guided nephrostomy in patients with non-obstructive uropathy due to urine leaks in cases of failed ultrasound-guided procedures. Eur J Radiol. 2011;80(3):686–91. 20971592 10.1016/j.ejrad.2010.09.035

[24] Hausegger KA, Portugaller HR. Percutaneous nephrostomy and antegrade ureteral stenting: technique-indications-complications. Eur Radiol. 2006;16(9):2016–30. 16547709 10.1007/s00330-005-0136-7

[25] de Baere T, Roche A, Lagrange C, Denys A, Court B, Isapoff J, et al. Combined percutaneous antegrade and cystoscopic retrograde approach in the treatment of distal ureteric fistulae. Cardiovasc Intervent Radiol. 1995;18(6):349–52. 8591619 10.1007/BF00338300

[26] Macri A, Magno C, Certo A, Basile A, Scuderi G, Crescenti F, et al. Combined antegrade and retrograde ureteral stenting: the rendezvous technique. Clin Radiol. 2005;60(2):257–60. 15664581 10.1016/j.crad.2004.06.008

[27] Mazzon G, Smith D, Arumuham V, Celentano G, Bolgeri M, Allen S, et al. Long-term outcomes of minimally invasive rendezvous procedures to treat complex ureteric strictures and injuries. Eur Urol Open Sci. 2023;49:53–9. 36874605 10.1016/j.euros.2022.12.014 PMC9974967

[28] Franco I, Eshghi M, Schutte H, Park T, Fernandez R, Choudhury M, et al. Value of proximal diversion and ureteral stenting in management of penetrating ureteral trauma. Urology. 1988;32(2):99–102. 3400148 10.1016/0090-4295(88)90306-8

[29] Toporoff B, Sclafani S, Scalea T, Vieux E, Atweh N, Duncan A, et al. Percutaneous antegrade ureteral stenting as an adjunct for treatment of complicated ureteral injuries: J Trauma. 1992;32(4):534–8. 1569628 10.1097/00005373-199204000-00019

[30] Mehra K, Manikandan R, Dorairajan LN, Sreenivasan Kodakkattil S, Kalra S. Effect of Ureteral Stent Length and Position of Stent Coil in Bladder on Stent-Related Symptoms and Quality of Life of Patients. Cureus. 2020;12(11):e11669. 33391907 10.7759/cureus.11669 PMC7769726

[31] Ho CH, Tai HC, Chang HC, Hu FC, Chen SC, Lee YJ, et al. Predictive factors for ureteral double-J-stent-related symptoms: a prospective, multivariate analysis. J Formos Med Assoc. 2010;109(11):848–56. 21126657 10.1016/S0929-6646(10)60130-1

[32] Patel U, Abubacker MZ. Ureteral stent placement without postprocedural nephrostomy tube: experience in 41 patients. Radiology. 2004;230(2):435–42. 14688404 10.1148/radiol.2302030078

[33] Pilcher JM, Patel U. Choosing the correct length of ureteric stent: a formula based on the patient’s height compared with direct ureteric measurement. Clin Radiol. 2002;57(1):59–62. 11798204 10.1053/crad.2001.0737

[34] Herrera M, Brawerman S, Castaneda WR, Kotula F, Amplatz K. The endocatheter ruler: a useful new device. AJR Am J Roentgenol. 1982;139(4):828–9. 6981950 10.2214/ajr.139.4.828

[35] Barrett K, Foell K, Lantz A, Ordon M, Lee JY, Pace KT, et al. Best stent length predicted by simple CT measurement rather than patient height. J Endourol. 2016;30(9):1029–32. 27338649 10.1089/end.2016.0105

[36] Kawahara T, Ito H, Terao H, Yoshida M, Ogawa T, Uemura H, et al. Which is the best method to estimate the actual ureteral length in patients undergoing ureteral stent placement? Int J Urol. 2012;19(7):634–8. 22435420 10.1111/j.1442-2042.2012.02998.x

[37] Elsamra SE, Leavitt DA, Motato HA, Friedlander JI, Siev M, Keheila M, et al. Stenting for malignant ureteral obstruction: tandem, metal or metal-mesh stents. Int J Urol. 2015;22(7):629–36. 25950837 10.1111/iju.12795

[38] Asakawa J, Iguchi T, Tamada S, Ninomiya N, Kato M, Yamasaki T, et al. Treatment outcomes of ureteral stenting for malignant extrinsic ureteral obstruction: a comparison between polymeric and metallic stents. Cancer Manag Res. 2018;10:2977–82. 30214292 10.2147/CMAR.S172283 PMC6118249

[39] Song Y, Fei X, Song Y. Diagnosis and operative intervention for problematic ureteral calculi during pregnancy. Int J Gynaecol Obstet. 2013;121(2):115–8. 23481356 10.1016/j.ijgo.2012.12.012

[40] Lai AL, Choong M, Toh L, Irani FG, Damodharan K, Chan S, et al. Radiological retrograde ureteric stent exchange in women: a single-centre review. Clin Radiol. 2020;75(6):480 e11-480 e16. 32156418 10.1016/j.crad.2020.02.003

[41] Carrafiello G, Coppola A, De Marchi G, Fontana F, Piacentino F, Petrillo M, et al. Trans-urethral ureteral stent replacement technique (TRUST): 10-year experience in 1168 patients. Cardiovasc Intervent Radiol. 2018;41(4):610–7. 29234836 10.1007/s00270-017-1854-3

[42] Ozkan O, Akinci D, Bozlar U, Ustunsoz B, Ozmen M, Akhan O. Retrograde ureteral stent exchange under fluoroscopic guidance. Diagn Interv Radiol. 2009;15(1):51–6. 19263375

[43] Smyth R, Mulholland D, Courtney M, Brennan I, McEniff N, Guiney M, et al. Retrograde ureteric stent exchange in the female oncology patient by interventional radiology: the experience of a single tertiary referral centre. Ir J Med Sci. 2020;189(3):1097–104. 32006389 10.1007/s11845-020-02170-1

[44] Halstuch D, Ehrlich Y, Shenhar C, Mano R, Baniel J, Margel D, et al. Transplant kidney retrograde ureteral stent placement and exchange: overcoming the challenge. Urology. 2018;111:220–4. 28965862 10.1016/j.urology.2017.09.012

[45] Tapping CR, Briggs JH, Little MW, Bratby MJ, Phillips-Hughes J, Crew JP, et al. Retrograde transileal conduit stent placement for obstructed uropathy--success of primary and exchange stent placement. J Vasc Interv Radiol. 2014;25(8):1250–6. 24698196 10.1016/j.jvir.2014.02.013

[46] Koral K, Saker MC, Morello FP, Rigsby CK, Donaldson JS. Conventional versus modified technique for percutaneous nephrostomy in newborns and young infants. J Vasc Interv Radiol. 2003;14(1):113–6. 12525597 10.1097/01.rvi.0000052301.26939.20

[47] Ozbek O, Kaya HE, Nayman A, Saritas TB, Guler I, Koc O, et al. Rapid percutaneous nephrostomy catheter placement in neonates with the trocar technique. Diagn Interv Imaging. 2017;98(4):315–9. 27765515 10.1016/j.diii.2016.08.010

[48] Cahill AM, Annam A, Baskin KM, Caplin D, Cramer HR Jr., Connolly B, et al. Society of interventional radiology quality improvement standards for percutaneous nephrostomy in the pediatric population. J Vasc Interv Radiol. 2021;32(1):146–9. 33388108 10.1016/j.jvir.2020.07.029

[49] Cyphers E, Gaballah M, Acord M, Worede F, Srinivasan A, Vatsky SE, et al. Percutaneous nephrostomy in neonates and young infants. J Vasc Interv Radiol. 2023;34(10):1815–21. 37336489 10.1016/j.jvir.2023.06.017

[50] Akand M, Sarica K, Kiremit MC, Soytas M, Guven S. Development of a prospective data registry system for retrograde intrarenal surgery in renal stones: Turkish Academy of Urology Prospective Study Group (ACUP study). Türk Üroloji Dergisi/Turkish Journal of Urology. 2020;46(1):57–62. 10.5152/tud.2019.19143 PMC6944417 31747366

[51] Thakur APS, Sharma V, Ramasamy V, Choudhary A, Patel, P, Singh S, Parol S. Management of ureteric stone in pregnancy: a review. African Journal of Urology. 2020;26(60).

[52] Xu K, Li J, Liu Y, Jiao D, Han X. Percutaneous nephroureteral stent placement and antegrade forceps biopsy of ureteral obstruction. J Vasc Interv Radiol. 2024;35(3):404–8. 37939999 10.1016/j.jvir.2023.10.032

[53] Pabon-Ramos WM, Dariushnia SR, Walker TG, d’Othee BJ, Ganguli S, Midia M, et al. Quality Improvement Guidelines for Percutaneous Nephrostomy. J Vasc Interv Radiol. 2016;27(3):410–4. 26803576 10.1016/j.jvir.2015.11.045

[54] Degirmenci T, Gunlusoy B, Kozacioglu Z, Arslan M, Ceylan Y, Ors B, et al. Utilization of a modified Clavien Classification System in reporting complications after ultrasound-guided percutaneous nephrostomy tube placement: comparison to standard Society of Interventional Radiology practice guidelines. Urology. 2013;81(6):1161–7. 23618426 10.1016/j.urology.2013.02.038

[55] Wah TM, Weston MJ, Irving HC. Percutaneous nephrostomy insertion: outcome data from a prospective multi-operator study at a UK training centre. Clin Radiol. 2004;59(3):255–61. 15037138 10.1016/j.crad.2003.10.021

[56] Miller GL, Summa J. Transcolonic placement of a percutaneous nephrostomy tube: recognition and treatment. J Vasc Interv Radiol. 1997;8(3):401–3. 9152913 10.1016/s1051-0443(97)70580-3

[57] LeRoy AJ, Williams HJ Jr, Bender CE, Segura JW, Patterson DE, Benson RC. Colon perforation following percutaneous nephrostomy and renal calculus removal. Radiology. 1985;155(1):83–5. 3975424 10.1148/radiology.155.1.3975424

[58] Traxer O. Management of injury to the bowel during percutaneous stone removal. J Endourol. 2009;23(10):1777–80. 19747058 10.1089/end.2009.1553

[59] Farrell TA, Hicks ME. A review of radiologically guided percutaneous nephrostomies in 303 patients. J Vasc Interv Radiol. 1997;8(5):769–74. 9314366 10.1016/s1051-0443(97)70658-4

[60] Sampaio FJ, Zanier JF, Aragao AH, Favorito LA. Intrarenal access: 3-dimensional anatomical study. J Urol. 1992;148(6):1769–73. 1433604 10.1016/s0022-5347(17)37024-6

[61] Radecka E, Magnusson A. Complications associated with percutaneous nephrostomies. A retrospective study. Acta Radiol. 2004;45(2):184–8. 15191103 10.1080/02841850410003671

[62] Chalmers N, Jones K, Drinkwater K, Uberoi R, Tawn J. The UK nephrostomy audit. Can a voluntary registry produce robust performance data? Clin Radiol. 2008;63(8):888–94. 10.1016/j.crad.2007.10.021 18625353

[63] Montvilas P, Solvig J, Johansen TE. Single-centre review of radiologically guided percutaneous nephrostomy using “mixed” technique: success and complication rates. Eur J Radiol. 2011;80(2):553–8. 21353753 10.1016/j.ejrad.2011.01.109

[64] Lee WJ, Patel U, Patel S, Pillari GP. Emergency percutaneous nephrostomy: results and complications. J Vasc Interv Radiol. 1994;5(1):135–9. 8136591 10.1016/s1051-0443(94)71470-6

[65] Lee WJ, Smith AD, Cubelli V, Badlani GH, Lewin B, Vernace F, et al. Complications of percutaneous nephrolithotomy. AJR Am J Roentgenol. 1987;148(1):177–80. 3491509 10.2214/ajr.148.1.177

[66] Kaskarelis IS, Papadaki MG, Malliaraki NE, Robotis ED, Malagari KS, Piperopoulos PN. Complications of percutaneous nephrostomy, percutaneous insertion of ureteral endoprosthesis, and replacement procedures. Cardiovasc Intervent Radiol. 2001;24(4):224–8. 11779010 10.1007/s00270-001-0004-z

[67] Ng CK, Yip SK, Sim LS, Tan BH, Wong MY, Tan BS, et al. Outcome of percutaneous nephrostomy for the management of pyonephrosis. Asian J Surg. 2002;25(3):215–9. 12376218 10.1016/S1015-9584(09)60178-0

[68] Bahu R, Chaftari AM, Hachem RY, Ahrar K, Shomali W, El Zakhem A, et al. Nephrostomy tube related pyelonephritis in patients with cancer: epidemiology, infection rate and risk factors. J Urol. 2013;189(1):130–5. 23164390 10.1016/j.juro.2012.08.094

[69] Monsky WL, Molloy C, Jin B, Nolan T, Fernando D, Loh S, et al. Quality-of-life assessment after palliative interventions to manage malignant ureteral obstruction. Cardiovasc Intervent Radiol. 2013;36(5):1355–63. 23404519 10.1007/s00270-013-0571-9 PMC4601638

[70] Saad WE, Virdee S, Davies MG, Patel NC, Sahler LG, Lee DE, et al. Inadvertent discontinuation of percutaneous nephrostomy catheters in adult native kidneys: incidence and percutaneous management. J Vasc Interv Radiol. 2006;17(9):1457–64. 16990465 10.1097/01-RVI.0000235703.68819.8C

[71] Geavlete P, Georgescu D, Multescu R, Stanescu F, Cozma C, Geavlete B. Ureteral stent complications - experience on 50,000 procedures. J Med Life. 2021;14(6):769–75. 35126746 10.25122/jml-2021-0352 PMC8811679

[72] Lennon GM, Thornhill JA, Sweeney PA, Grainger R, McDermott TE, Butler MR. “Firm” versus “soft” double pigtail ureteric stents: a randomised blind comparative trial. Eur Urol. 1995;28(1):1–5. 8521886 10.1159/000475010

[73] Joshi HB, Stainthorpe A, MacDonagh RP, Keeley FX Jr, Timoney AG, Barry MJ. Indwelling ureteral stents: evaluation of symptoms, quality of life and utility. J Urol. 2003;169(3):1065–9. 12576847 10.1097/01.ju.0000048980.33855.90

[74] el-Faqih SR, Shamsuddin AB, Chakrabarti A, Atassi R, Kardar AH, Osman MK, et al. Polyurethane internal ureteral stents in treatment of stone patients: morbidity related to indwelling times. J Urol. 1991;146(6):1487–91. 1942324 10.1016/s0022-5347(17)38146-6

[75] Kram W, Buchholz N, Hakenberg OW. Ureteral stent encrustation. Pathophysiology. Arch Esp Urol. 2016;69(8):485–93. 27725325

[76] Kawahara T, Ito H, Terao H, Yoshida M, Matsuzaki J. Ureteral stent encrustation, incrustation, and coloring: morbidity related to indwelling times. J Endourol. 2012;26(2):178–82. 22007916 10.1089/end.2011.0385

[77] Nerli RB, Magdum PV, Sharma V, Guntaka AK, Hiremath MB, Ghagane S. Forgotten/retained double J ureteric stents: a source of severe morbidity in children. Afr J Paediatr Surg. 2016;13(1):32–5. 27251521 10.4103/0189-6725.181704 PMC4955456

[78] Agrawal M, Gite VA, Sankapal P, Maheshwari M, Shah A, Dias S, et al. Retained ureteral stents, an avoidable source of morbidity: 10 years experience from a single tertiary care centre. Pan Afr Med J. 2022;42:68. 35949469 10.11604/pamj.2022.42.68.29935 PMC9338717

[79] Pappas P, Stravodimos KG, Mitropoulos D, Kontopoulou C, Haramoglis S, Giannopoulou M, et al. Role of percutaneous urinary diversion in malignant and benign obstructive uropathy. J Endourol. 2000;14(5):401–5. 10958560 10.1089/end.2000.14.401

[80] Zul Khairul Azwadi I, Norhayati MN, Abdullah MS. Percutaneous nephrostomy versus retrograde ureteral stenting for acute upper obstructive uropathy: a systematic review and meta-analysis. Sci Rep. 2021;11(1):6613. 33758312 10.1038/s41598-021-86136-y PMC7988020

[81] Xu ZH, Yang YH, Zhou S, Lv JL. Percutaneous nephrostomy versus retrograde ureteral stent for acute upper urinary tract obstruction with urosepsis. J Infect Chemother. 2021;27(2):323–8. 33309627 10.1016/j.jiac.2020.11.022

[82] Assimos D, Crisci A, Culkin D, Xue W, Roelofs A, Duvdevani M, et al. Preoperative JJ stent placement in ureteric and renal stone treatment: results from the Clinical Research Office of Endourological Society (CROES) ureteroscopy (URS) global study. BJU Int. 2016;117(4):648–54. 26237735 10.1111/bju.13250

[83] Fonio P, Appendino E, Calandri M, Faletti R, Righi D, Gandini G. Treatment of urological complications in more than 1,000 kidney transplantations: the role of interventional radiology. Radiol Med. 2015;120(2):206–12. 25027279 10.1007/s11547-014-0407-y

[84] Postoak D, Simon JM, Monga M, Ferral H, Thomas R. Combined percutaneous antegrade and cystoscopic retrograde ureteral stent placement: an alternative technique in cases of ureteral discontinuity. Urology. 1997;50(1):113–6. 9218029 10.1016/s0090-4295(97)00204-5

[85] Horer TM, Ierardi AM, Carriero S, Lanza C, Carrafiello G, McGreevy DT. Emergent vessel embolization for major traumatic and non-traumatic hemorrhage: indications, tools and outcomes. Semin Vasc Surg. 2023;36(2):283–99. 37330241 10.1053/j.semvascsurg.2023.04.011

[86] de Baere T, Denys A, Pappas P, Challier E, Roche A. Ureteral stents: exchange under fluoroscopic control as an effective alternative to cystoscopy. Radiology. 1994;190(3):887–9. 8115645 10.1148/radiology.190.3.8115645

[87] Frederick L, Ellimoottil C, Kadlec A, Shah A, Turk T, Schwartz BF. Cost analysis of metallic stents for chronic ureteral obstruction: a multicenter study. Urol Pract. 2017;4(1):21–4. 37592614 10.1016/j.urpr.2016.03.008

[88] Pappas P, Stravodimos KG, Kapetanakis T, Leonardou P, Koutallelis G, Adamakis I, et al. Ureterointestinal strictures following Bricker ileal conduit: management via a percutaneous approach. Int Urol Nephrol. 2008;40(3):621–7. 18320342 10.1007/s11255-008-9349-4

[89] Alago W Jr., Sofocleous CT, Covey AM, Thornton RH, Donat SM, Brody LA, et al. Placement of transileal conduit retrograde nephroureteral stents in patients with ureteral obstruction after cystectomy: technique and outcome. AJR Am J Roentgenol. 2008;191(5):1536–9. 18941097 10.2214/AJR.08.1003

